# Groin Hernia Repair, the History of the Open Pre-Peritoneal Route Towards a Minimally Invasive Approach. Narrative Review

**DOI:** 10.3389/jaws.2025.13861

**Published:** 2025-02-17

**Authors:** Marc Soler

**Affiliations:** Clinique Saint Jean, Cagnes Sur Mer, France

**Keywords:** prosthesis, groin hernia, history, open, preperitoneal

## Abstract

The history of pre peritoneal groin hernia surgery start only after solving the problems related to asepsis, antisepsis and anesthesia. Fundamental work on the use of a new form of polyethylene to create synthetic meshes was carried out in the 1950s by C. Usher. L. Nyhus was the first to popularize the use of a mesh. But the inventor of the first synthetic prosthesis was Don Eugène Acquaviva in 1944, and the first surgeon to discuss the installation of a pre-peritoneal prosthesis for the treatment of hernias of the groin is Jerome Corti in his thesis in 1949. In the 50 s and 60 s H. Fruchaud had particularly and directly influenced Jean Rives and René Stoppa, and due to the poor results of techniques without prosthesis, particularly for complex hernias Rives and Stoppa techniques were then disseminated with lots of variations, (G. Wantz, J.H. Alexandre, R. D. Kugel….) But the parietalization step was difficult to achieve for many colleagues and the development of endoscopy has made it possible to clearly demonstrate this crucial step in order to properly unroll the prosthesis. Franz Ugahary put up resistance against endoscopy with the Grid Iron technique in 1995, the fist open minimal invasive pre peritoneal approach. In 2004, Pelissier invented a specific semi-rigid prosthesis, which made it possible to codify with colleagues the Trans Inguinal Pre-Peritoneal (TIPP) technique. But it was also necessary to master the step of parietalization of the cord, this is probably why the ONSTEP technique was created in 2005. It is a partially preperitoneal technique without parietalization W. Akkersdick has tackled the challenge with the Trans Rectus sheath Pre Peritoneal (TREPP) technique in 2006, a pure posterior approach. For my part I modified the TIPP technique in 2011 using Ugahary’s dissection principles, the Minimal Open Pre Peritoneal (MOPP) technique was created. It is only in recent years that the literature has provided data about TIPP, TREPP, MOPP, with comparisons with others techniques. Now the new route, preperitoneal, minimal open and minimal invasive has its place in the treatment of groin hernias!

## Introduction

Since antiquity, the history of inguinal hernia surgery is rich in anecdotes with most often fatal conclusions for patients [1], and this continues even during the second half of the 19th century where the subject of this article, dedicated to the preperitoneal approach to treat inguinal hernias, begins. The 19th century brings knowledge in terms of anatomy and in terms of hygiene to allow the surgeon to penetrate the preperitoneal space, the 20th century brings the synthetic prostheses to gain in efficiency, and finally the end of the 20th century and the beginning of the 21st century are decisive to define resolutely minimally invasive techniques on the basis of classical procedures. This new way then expresses itself fully by giving the first scientific guarantees that we are entitled to expect. The main objective of this article, besides recalling historical facts by sometimes correcting certain injustices concerning underestimated and sometimes even forgotten authors, is to put into perspective the links which exist between surgeons of several generations who had the same ambition, to perform a surgery that was *a priori* complex in the least invasive way possible and which was aimed at the greatest number of patients.

## Development

**FIGURE 1:**
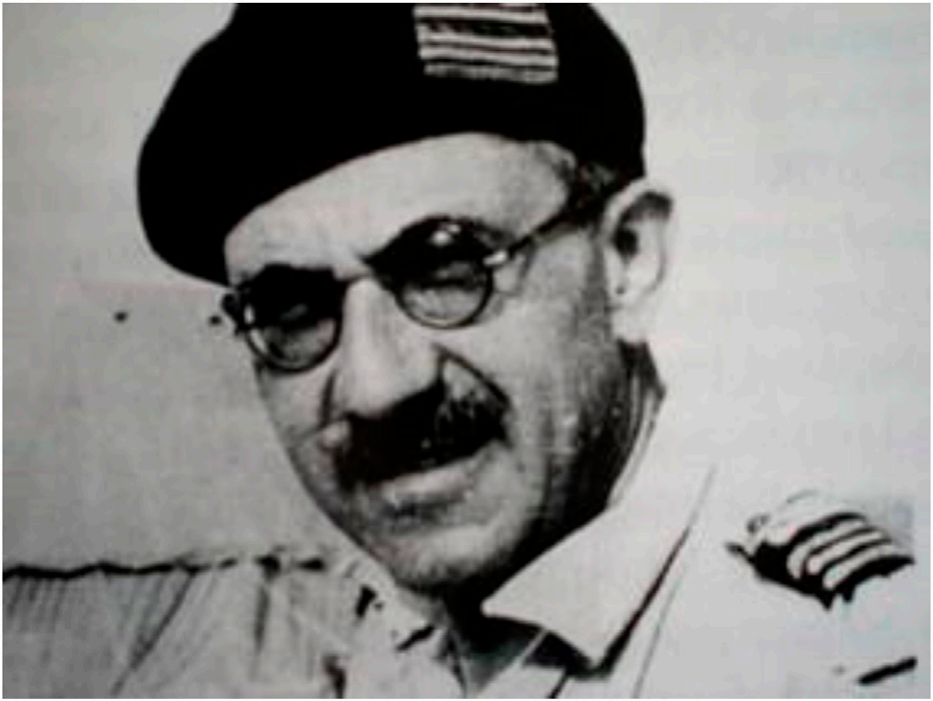
Henri Fruchaud (1894–1960).

The anatomical data were clarified and shared by A.P. Cooper in 1807 [2], A. Bogros in 1823 [3], A Thomson in 1836 [4], A.A. Retzius, 1858 [5], completed by E. Bassini in 1887 [6], and later by E.E. Shouldice in 1945 [7], H. Fruchaud in 1956–1957 [8, 9] (Figure 1), C.B. McVay in 1958 [10] and R.E. Condon in 1971 [11]. Even though knowledge of anatomy might have allowed it, therapeutic means were very ineffective before the use of asepsis, antisepsis and anesthesia. Thus, during the first part of the 19th century, the pioneers were mainly interested in these patients in very poor condition with an irreducible hernia whose outcome was in any case spontaneously fatal. They also used the posterior approach, but most often patients died of gangrene [1]. Most other patients who were not directly at risk for complications were not operated on.

During the same decades, the British surgeon Joseph Lister described in 1867 the success of a method to combat postoperative infections: antisepsis [12]. This idea came to him from the demonstration made by Louis Pasteur a few years before, which highlighted the role of microbes in the origin of infections. Asepsis came to complement Lister’s antisepsis, which had only been accepted very gradually. Ultimately, the two processes allowed a real development of surgery from 1885, and particularly allowed the opening of parietal spaces with a drastically reduced rate of fatal post-operative infection [1].

It is precisely around these same decades that research concerning local and general anesthesia would be published and spread [13]. From nitrous oxide to chloroform in the 1840s for general anesthesia, cocaine for local anesthesia, Freud 1884, P. Reclus (7,000 cases) Paris, and the invention of the spinal anesthesia in August 1898 by August Bier. All the pillars (anatomy, anesthesia, antisepsis, asepsis) were in place and would be immediately used for hernia surgery from the second part of the 18th and beginning of the 19th century.

The pioneers for the posterior approach are, according to Chavasse, Crompton of Birmingham [14], followed by Niven [15] and Annandale who repeated the Crompton’s procedure in 1876 [16]. He was followed by Lawson Tait [17] from Birmingham, then by Bates [18] and G.L. Cheatle 1920 from the England- King’s college hospital London, who was a devoted disciple of Lister [19]. Patino clarifies: “Cheatle, in 1920, described an operation for the radical cure of inguinal and femoral hernias through a medial abdominal section, without entering the peritoneal cavity” [20, 21], and in 1921 Cheatle reported on the use of the Pfannenstiel incision. In 1936 Henry [22] emphasized the advantages of Cheatle’s approach in the cure of bilateral femoral hernias with a little impact before World War II. At this period, we spoke about the Cheatle-Henry procedure that provides excellent exposure of anatomic structures adjacent to the femoral canal. And it is finally Henry who popularized the posterior approach among the pioneers of the second half of the 20th century, with Mc Evedy [23], but always without the help of a prosthesis. The gold standard at that time was the anterior approach, following the works of Bassini [6] and Shouldice [7]. For femoral hernias, McVay described his eponymous technique in 1938 [10], but surgeons did not accept his original description. They omitted making the relaxation incision and results were not as good as those published by McVay. So, everything was in place for the next decisive step which was the birth of synthetic prostheses.

**FIGURE 2:**
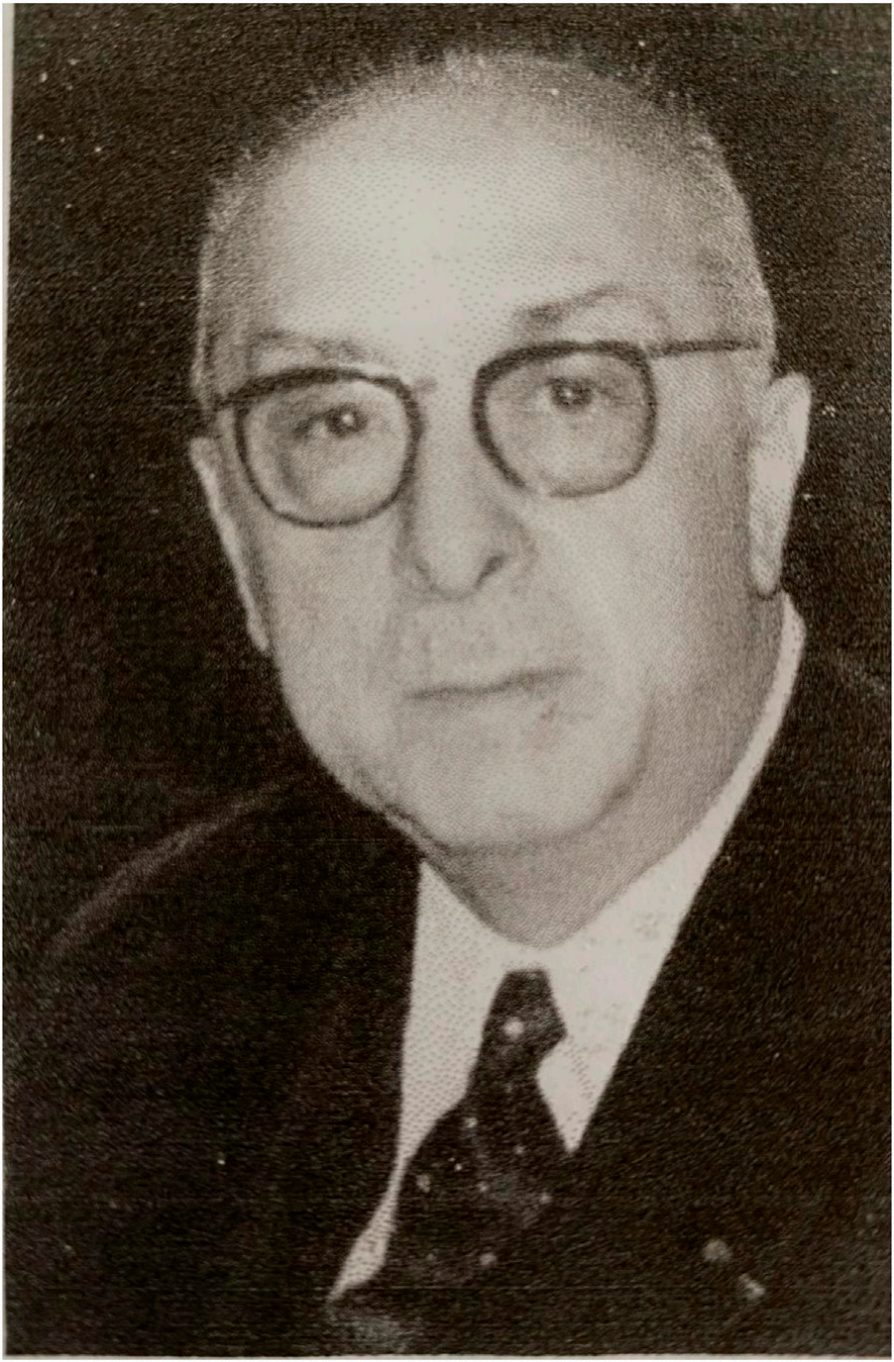
Don Eugène Acquaviva (1897–1976).

**FIGURE 3:**
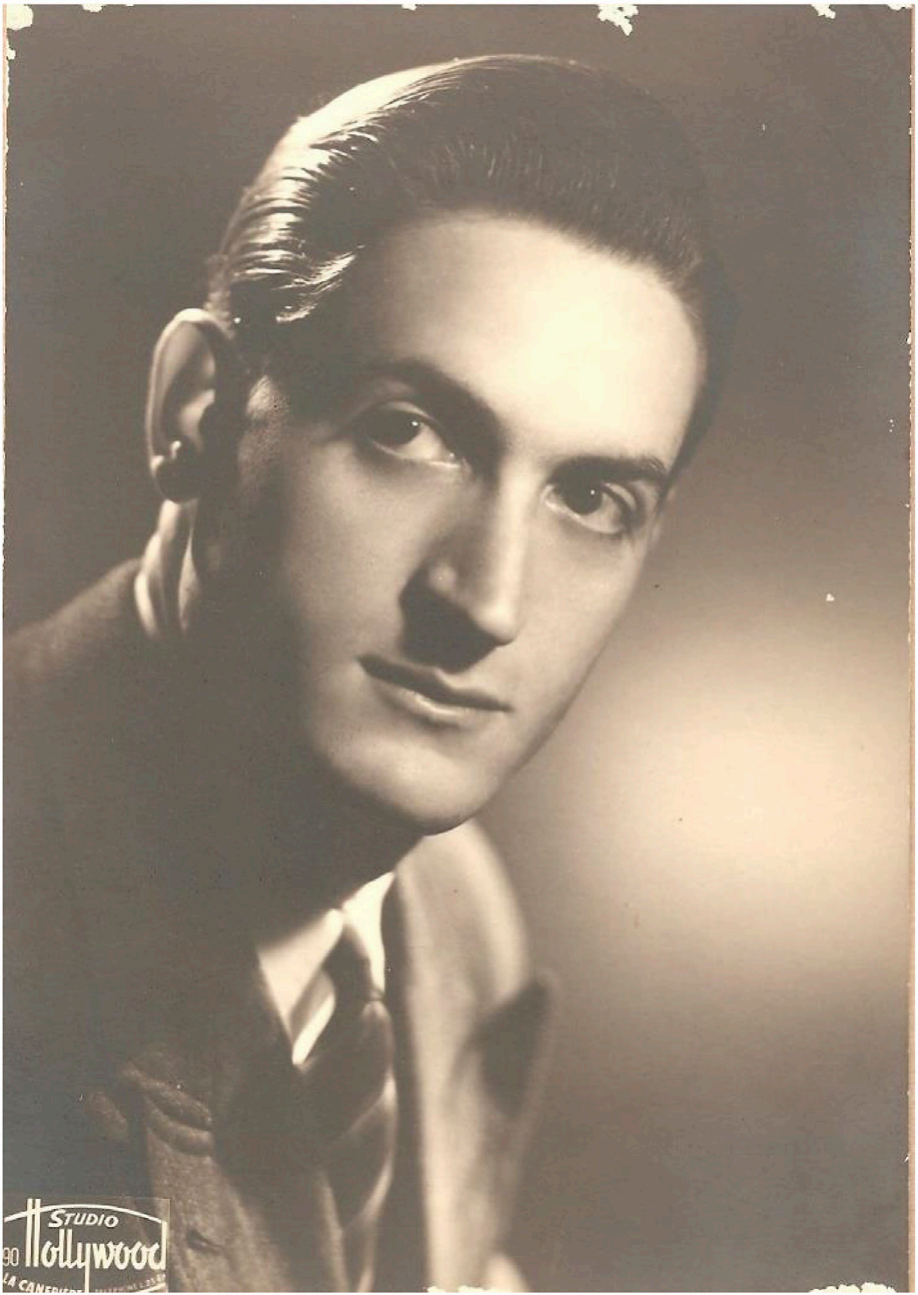
Dominique Corti (1919–1983).

The inventor of the first synthetic prosthesis was Don Eugène Acquaviva from Marseille. He personally had manufactured, patented and used a nylon mesh for an incisional hernia in 1944 [24, 25] (Figure 2), and the first surgeon to discuss the installation of a pre-peritoneal prosthesis for the treatment of a groin hernia, which was a femoral hernia (but without realizing it himself), was his son-in-law Dr Jérôme Corti in his thesis in 1949 [26] (Figure 3). Don Eugène Acquaviva was particularly innovative in terms of the design of synthetic mesh and its use in ventral surgery. His work was published thanks to the interest shown by Lucien Leger from Hospital Cochin in Paris, who was the editor of the “notes of surgical techniques” in the “Presse Medicale” journal. These notes were widely distributed and were serious references at the French as well as at the international level. The nylon prosthesis patented by Acquaviva would soon be used by Bourgeon and would also be inserted into the preperitoneal space [27, 28].

**FIGURE 4:**
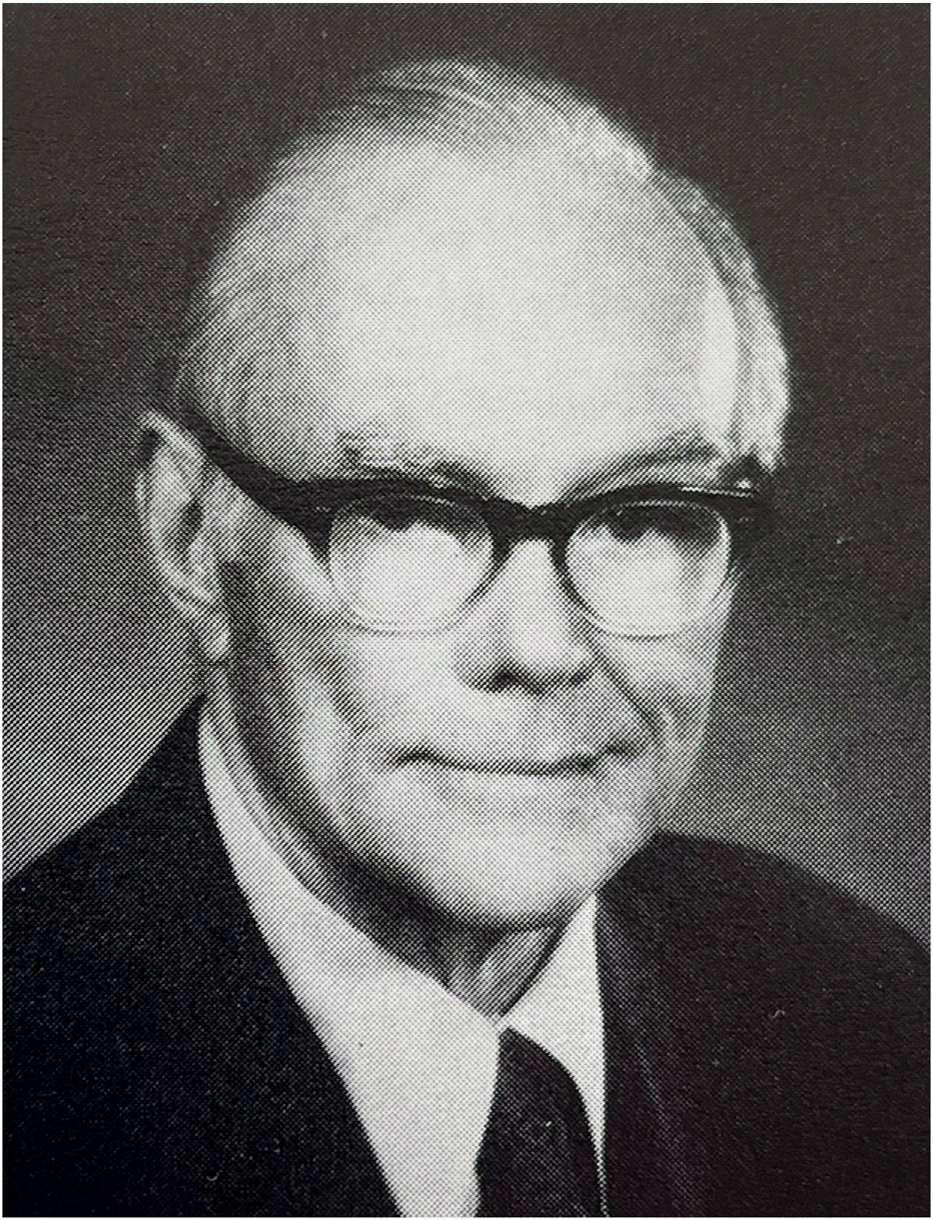
Francis C. Usher (1908–1980).

However, the fundamental work was carried out by C. Usher (Figure 4) in his private practice in Houston, Texas [29]. He used a new ethylene polymer woven into a mesh, the Marlex prosthesis, which was fabricated to his design, and used for groin hernia surgery in 1958. A knitted Marlex product was introduced in 1961. The same year a braided Marlex suture appeared [30]. Polypropylene monofilament, an isotactic polymer which retains its tensile strength, was introduced in 1962, recommended as an inert suture to close contaminated wounds [31, 32]. Monofilament polypropylene suture is still the preferred synthetic material today! Usher therefore carried out numerous experimental and clinical studies resulting in 20 papers on hernia between 1958 and 1967 [30–38]. As Read said [29], “Usher realized Billroth’s vision,” (1878) as quoted by Vincenz Czerny in his textbook, “If we could artificially produce tissues of the density and toughness of fascia and tendon, the secret of the radical cure of hernia would be discovered.”

**FIGURE 5:**
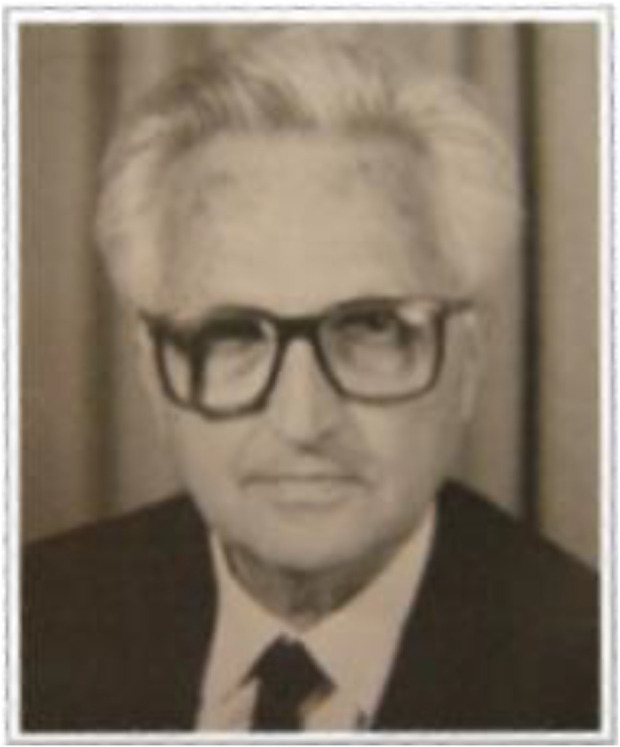
Jean Rives (1922–2012).

But the acceptance of prosthetic materials in parietal surgery was very low. As Read reminds us [39] Usher said, “surgeons are usually reluctant to use a prosthesis for fear of wound complications and a natural disinclination to use foreign materials.” Note that before Usher, and thanks to his own experimental work [31], early polymers such as Nylon (Acquaviva), Dacron, Orlon and Teflon had been the most studied, but the results were disappointing. Foreign body reaction, sepsis, stiffness, fragmentation, loss of tensile strength, and encapsulation have prevented their widespread use. Metal prostheses (Tantalum Gauze) had also given disappointing results, as Debord recalls [40], even though stainless-steel mesh had enthusiastic users until the 80s [41]. This hostility against protheses would last a long time and this was confirmed to me by Jean Rives (Figure 5). He told me during a 2-h recorded interview on 6 October 2011, that he had been heavily criticized in the 1960s and 1970s, because of his practice of using prosthetic material for simple non-recurrent hernias, even by his colleagues who were also his friends [42].

**FIGURE 6:**
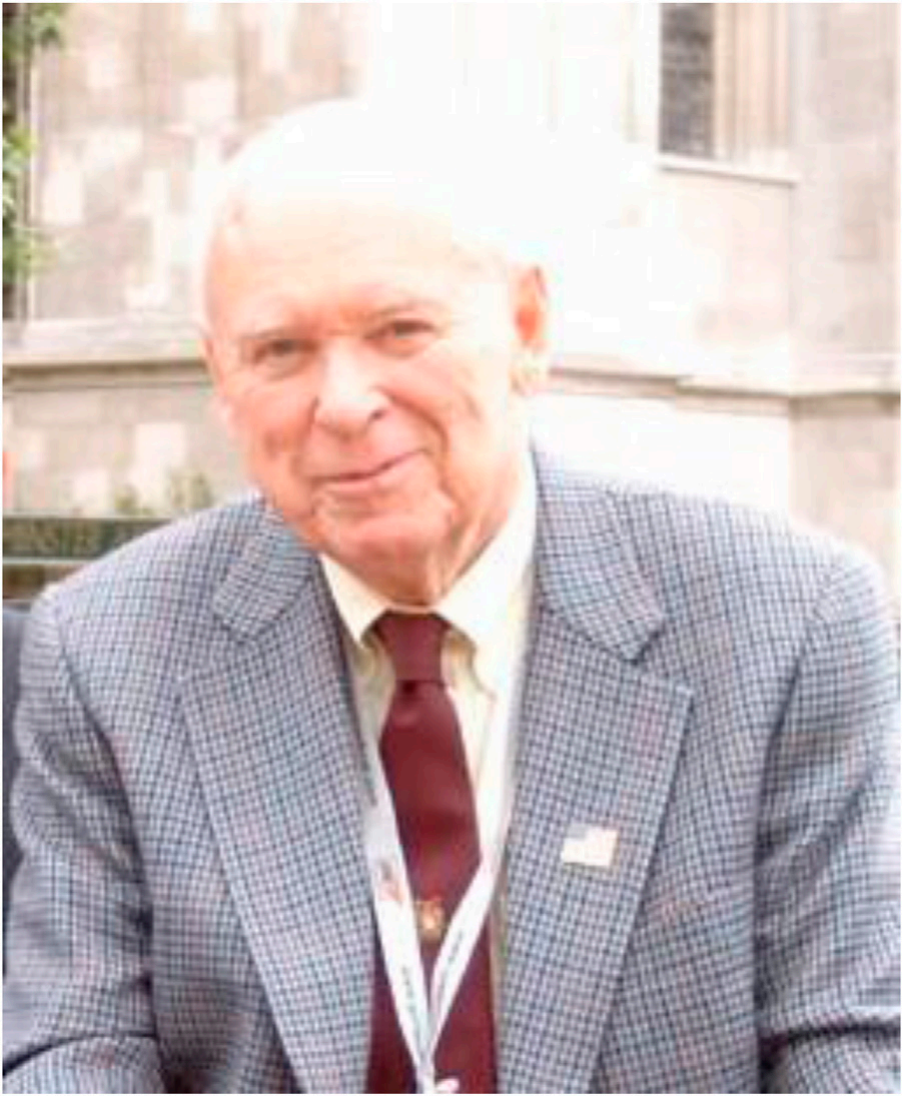
Llyod Milton Nyhus (1923–2008).

Moreover, even though Usher widely used prostheses in the preperitoneal space with parietalization of the cord, the official laurels would preferentially go to Nyhus (Figure 6) and too few colleagues who popularized the use of a mesh allowed the Cheatle-Henry and Mc Evedy incision [19–21]. In their 1959 paper, Nyhus and colleagues described the use of a synthetic sponge (Ivalon^®^) to reinforce the posterior wall of a recurrent hernia [43, 44]. Due to its poor tolerance, the Ivalon sponge would quickly be abandoned in favor of the Marlex mesh created by Usher. At that time, many surgeons applied the principles published by Nyhus: Sheehan (1961), Mahorn and Goss (196, Smith (1962), Huguier (1963), Estrin (1963), Andrews (1968) and Read (1968).

**FIGURE 7:**
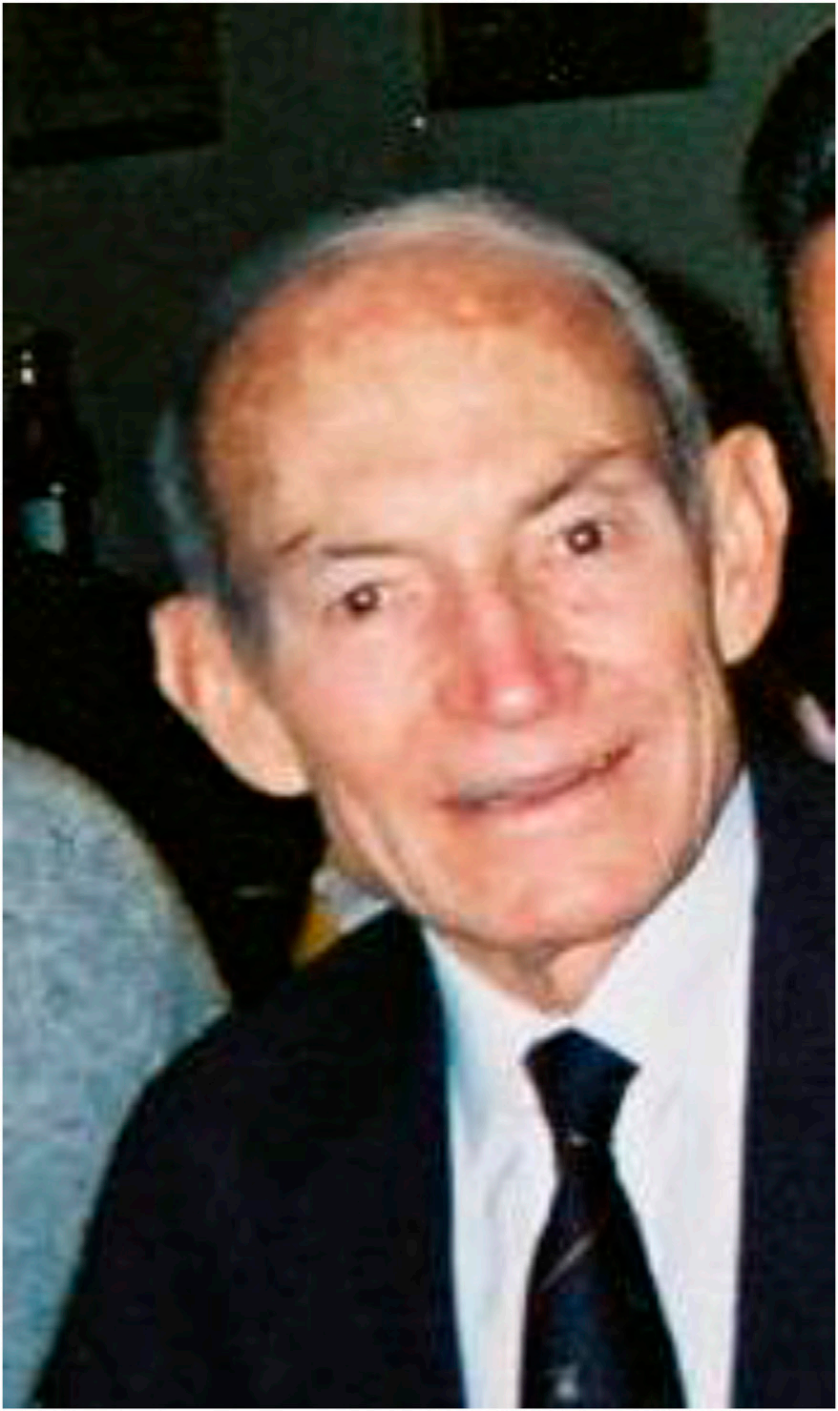
René Stoppa (1921–2006).

**FIGURE 8:**
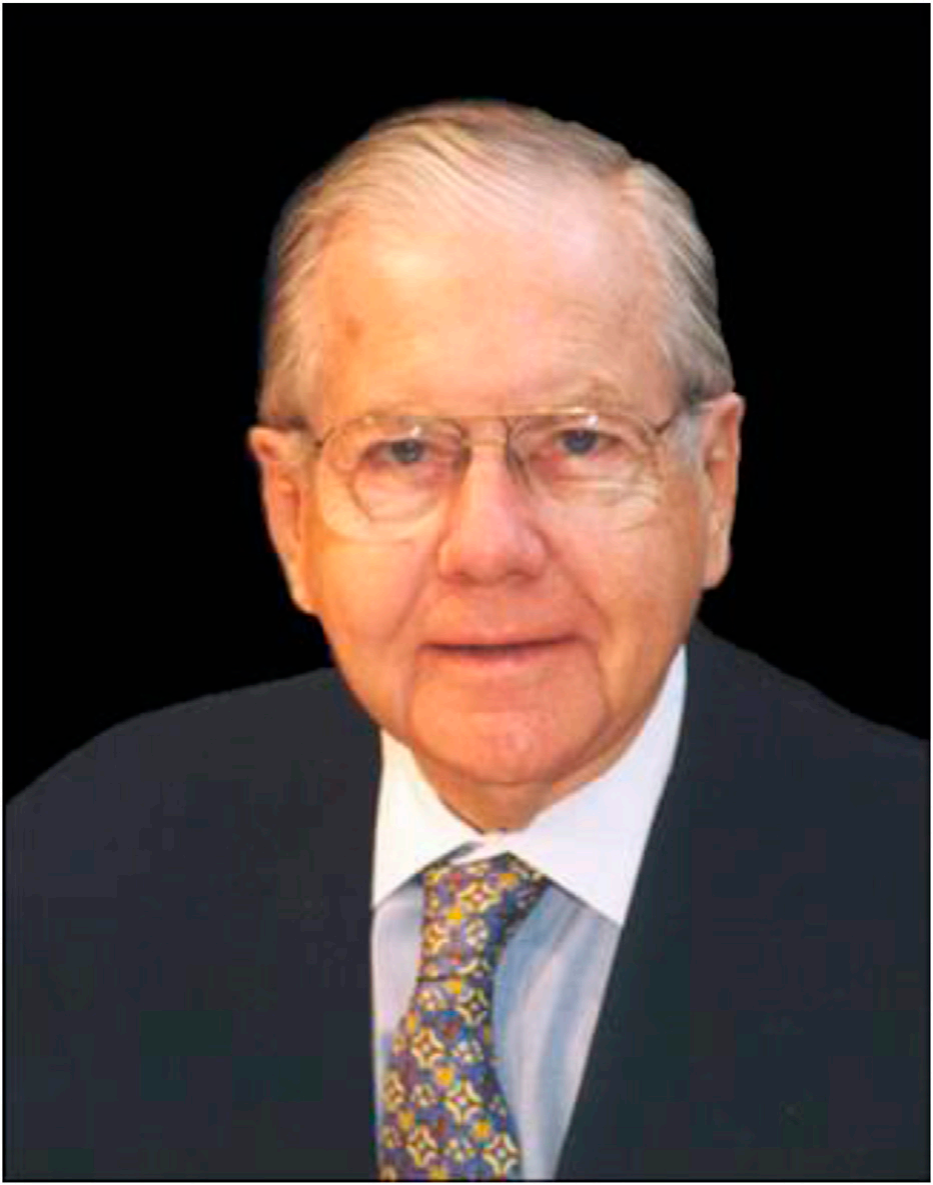
George Wantz (1923–2000).

**FIGURE 9:**
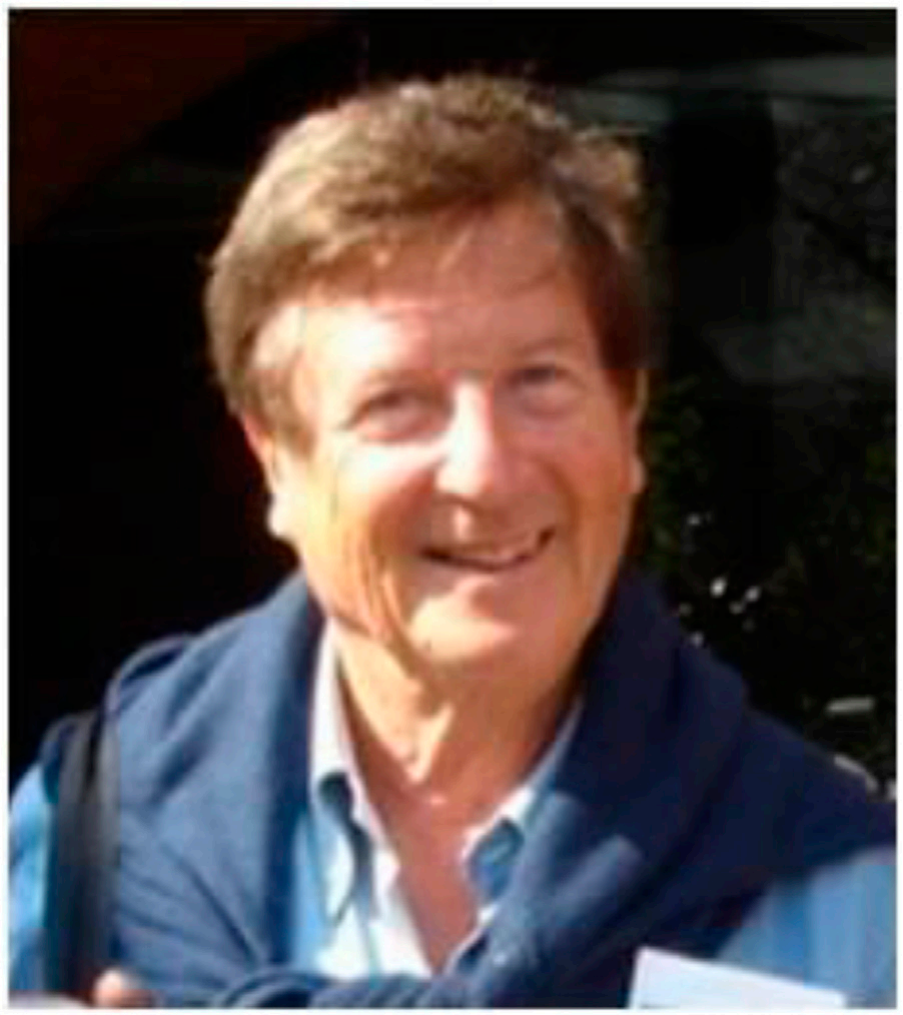
Jean Henry Alexandre (1931–2019).

In 1956, only a few years before Usher Nyhus and Henry, Fruchaud [9] had insisted on the need for broad coverage of the musculo-pectineal orifice which bears his name. Henry Fruchaud had particularly influenced Jean Rives and René Stoppa (Figure 7) who had worked in the anatomy laboratory of the Faculty of Medicine of Algiers. During this period Fruchaud worked on his two famous books [8, 9], never translated before Robert Bendavid in 2006 [45] and therefore largely unknown in the Anglo-Saxon world despite the efforts of R. Stoppa to promote them. So, in the 70s, directly influenced by their mentor, J. Rives [46, 47], and then R. Stoppa [48–50] described their techniques in a general context still unfavorable to the use of parietal prostheses, except gradually for complex groin hernias. The polyester Dacron mesh (mersilene) was first used in France by J. Rives following the presentation of the mesh by the laboratory that marketed it [41]. R. Stoppa was inspired by the Rives’ technique for bilateral hernias by creating his eponymous technique. Eventually, and probably partly due to the popularity of Lichtenstein’s technique, the gold standard in the 1980s, the idea of using a prosthesis had finally become commonly accepted. Even surgeons who were still against the use of prostheses admitted the advantages for the most complex cases, and the techniques of Rives and Stoppa were then disseminated with lots of variations, such as G. Wantz [51], (Figure 8), and J.H. Alexandre [52, 53], (Figure 9) techniques.

Jean Henri Alexandre’s technique is a step to remember towards less invasive solutions. It could be considered as a precursor of the TIPP technique. The approach was a classic anterior approach as it provided for the ligation of the inferior epigastric vessels to facilitate access to the preperitoneal plane, the prosthesis being fixed. But at the time these techniques required regional or general anesthesia and classic hospitalization.

**FIGURE 10:**
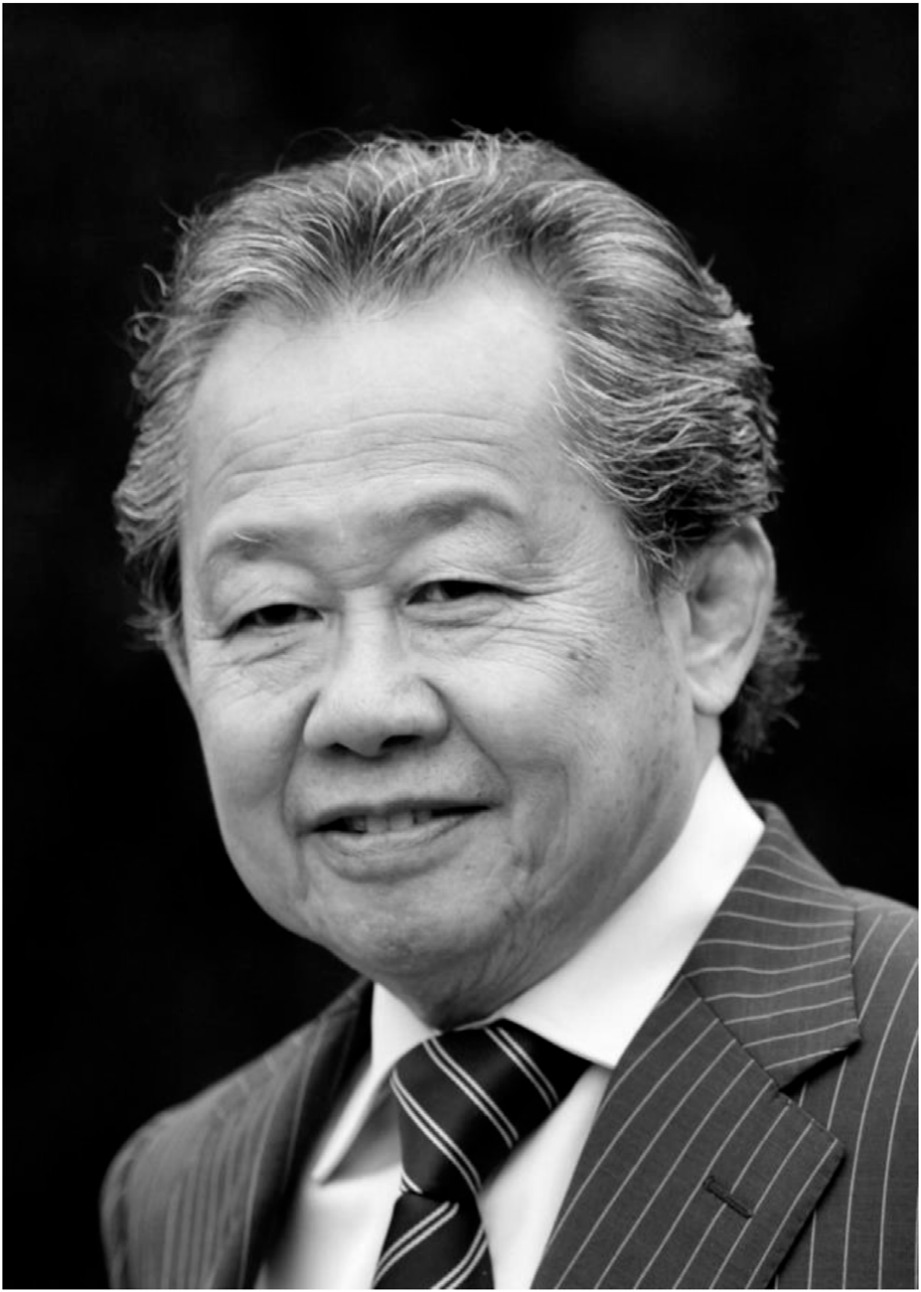
Franz Ugahary.

**FIGURE 11:**
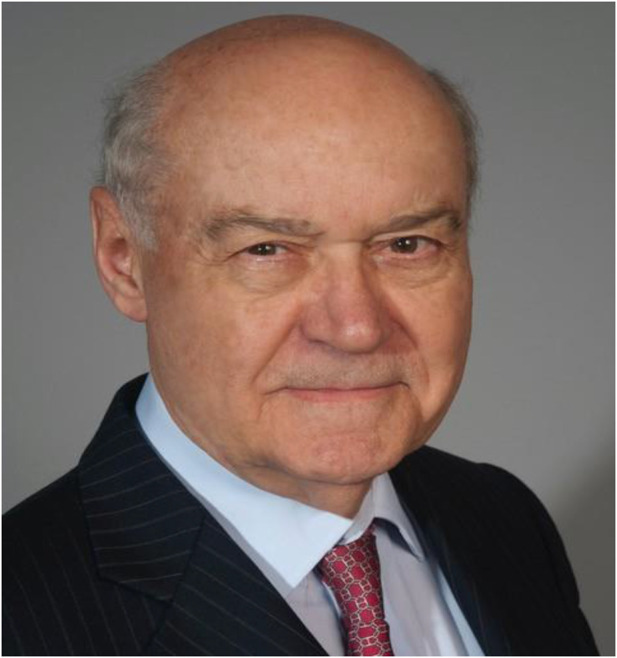
Edouard Pelissier.

**FIGURE 12:**
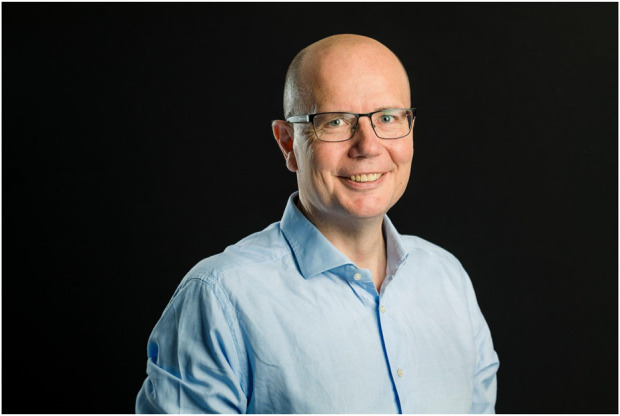
Frederik Berrevoet.

**FIGURE 13:**
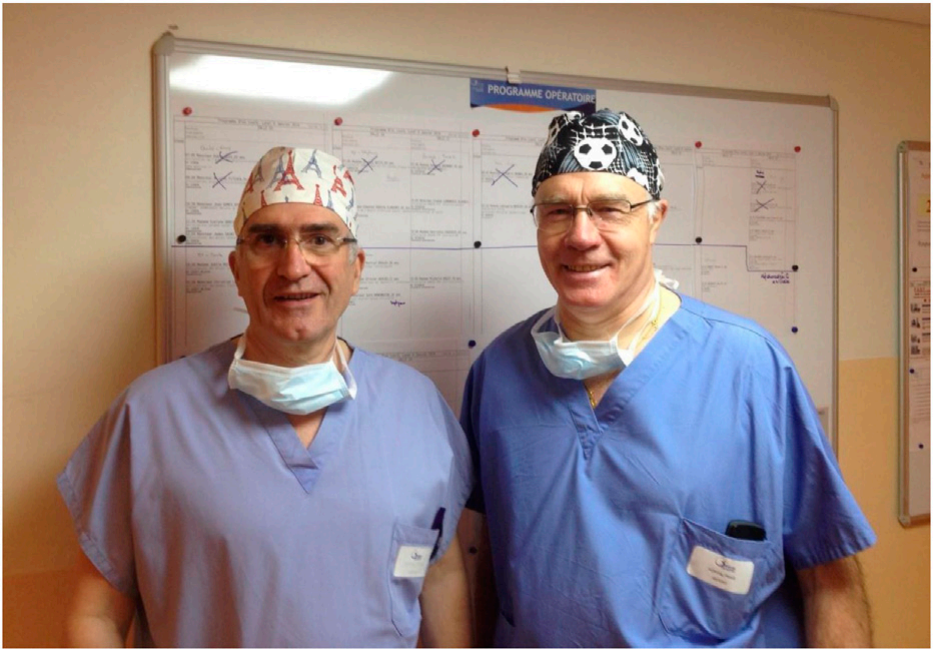
Jean François Gillion and Jean Michel Chollet.

Another variation is the R.D. Kugel technique [54]. The initial Kugel mesh had an abundant amount of foreign material present. Problems with the initial recoil ring resulted in pain and even bowel perforation. Another version of this mesh type contained a resorbable memory ring. The Kugel technique has often been compared to the TIPP technique [55]. This is where Franz Ugahary [56, 57] (Figure 10) and Edouard Pelissier [58] (Figure 11) come in and are seen as the two true pioneers of minimally invasive preperitoneal surgery: During the early 1990s they were thinking about how to place a prosthesis in the preperitoneal space in a decidedly less invasive way than the Stoppa and Rives techniques, used for complex cases by these authors and without wanting to embark on endoscopic surgery, which they found more invasive. In this period the TAPP was the most used endoscopic technique. Pelissier was convinced and familiar with techniques using the anterior route (modified Bassini, Shouldice) under local anesthesia, with the desire to promote outpatient care [59, 60]. He used the Stoppa Rives procedure for the more complex cases. In May 1990 he had the idea of using a pre-peritoneal prosthesis after hearing Gilbert [61] presenting his plug [62] at a conference in Nice during the first French international hernia surgery symposium. The plug was a 5 cm square of polypropylene opened from the middle of one side to the center and inserted through the deep inguinal opening, with the cord passing through the slit. However, the technique was not easy to achieve and there were not many indications. The second influence came from Rutkow and Robbins [63] who had designed the plug, which was introduced through the hernial orifice, but the prosthesis was not spread flat. Although the results seemed very good in terms of recurrence, it came at the cost of a rate of chronic pain (8.6%) due to the shrinkage of the prosthesis, which ended up forming a sort of hard core [64]. These two relative failures confirmed our pioneer in his first idea; to invent a prosthesis that spreads flat in the pre-peritoneal space, introduced through the hernia orifice, self-deploying, and by performing an intervention preferably under local anesthesia. Local anesthesia was associated with an outpatient procedure at this period, and already widely practiced in many countries, but unfortunately not in several others where it was almost impossible for reasons of organization of the health system, including France. But the idea of outpatient surgery was still on the minds of many. Pelissier began to work extensively from 1999 with the development of different prototypes which finally led in September 2004 to the launch of the first prosthesis specifically dedicated to being spread forward in the pre-peritoneal space: the Polysoft prosthesis. The prosthesis would first be split to allow passage for the cord [58], using the principle of the split prosthesis as in the Gilbert and Liechtenstein procedures, which was easier. Very quickly interested in Pelissier’s principle, Frederik Berrevoet (Figure 12) and Stephen de Gendt had invited E. Pelissier to the University Hospital of Ghent for a workshop. Frederik Berrevoet was not satisfied with Lichtenstein’s technique which was mainly used in Belgium at that time and not convinced by the intraperitoneal route of the TAPP endoscopic technique which was spreading more quickly than the TEP technique. They immediately used the technique with the specific prosthesis but without splitting he prosthesis and therefore parietalizing the cord [65, 66]. And Pelissier would quickly make the parietalization, because of two recurrences through the split in the prosthesis [61]. It is very interesting to point out that the technique was thus finalized by E. Pelissier, F. Berrevoet, S. De Gendt and colleagues in Belgium and quickly disseminated in France by J.F. Gillion and J.M. Chollet [67, 68] (Figure 13) and finally called Trans Inguinal Pre-Peritoneal (TIPP). It still appeared difficult to achieve for certain colleagues who returned to the Lichtenstein technique, after having learned the technique from the first promoters who organized workshops in their respective operating rooms, as observed by E. Pelissier [61]. And for the same reason two authors rom Porto, A. Lourenco and R. S. da Costa, developed the Onstep technique in 2005, pursuing the idea of splitting the prosthesis and simplifying the learning of the technique by avoiding the parietalization step [69]. Onstep technique is a partially preperitoneal technique as the lower and medial part of the prosthesis are in the preperitoneal space and the upper and lateral part are positioned as in the Lichtenstein technique, under the aponeurosis of the external oblique muscle.

**FIGURE 14:**
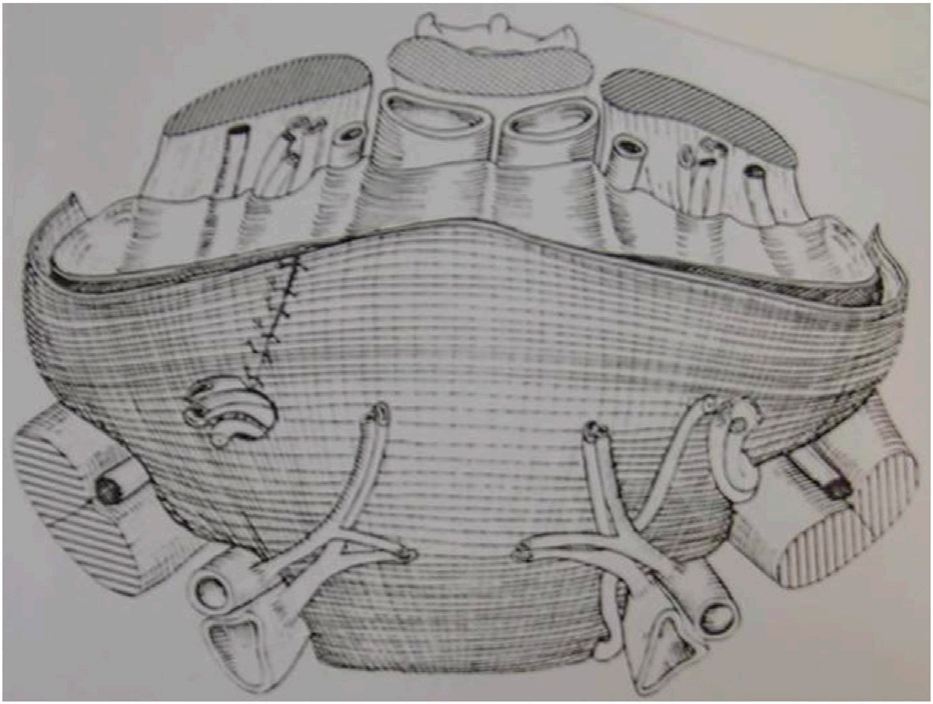
Stoppa: The great prosthesis for the reinforcement of the visceral sac (GPRVS).

We can thus realize that in this adventure one of the points of friction remains the notion of parietalization ! Let’s look at this:

Due to the not the experience of surgeons, this step of parietalization has slowed down the dissemination of the posterior route. This is why René Stoppa in 1973 published the drawing of his intervention “the Giant Preperitoneal Repair” (GPPR), one side with parietalization and the other side with a slit in the prosthesis even though in his practice he never split the prosthesis (Figure 14). If Acquaviva [24, 25] and Bourgeon [27, 28] were pionners to develop the principle of spreading a prosthesis in the preperitoneal space, it was Usher, who was the first in 1959 to publish the principle of parietalization of the cord as Read recalls [29]. Read said: “Another valuable concept he (Usher) documented was the use of unsplit groin prostheses with overlap and interrupted suturing lateral to the internal inguinal ring to allow extended preperitoneal obliquity of the spermatic cord. In his own (Usher) words: “Rather than cut a notch in the mesh, we prefer to suture the lateral border of the mesh well lateral to the curving border of the internal oblique muscle, providing a 'shelf’ for the cord to rest on, and preserving the normal obliquity of the internal ring.” Here is yet another reason to highlight Usher, this formidable precursor who epitomizes the best of 20th Century Herniology” [29]. In 1992, Jean Henri Alexandre was the first to propose the parietalization of the cord by an anterior inguinal incision, making his technique an early version of TIPP [52, 53]. Everything accelerated with the arrival of endoscopic surgery during the early 90s, with very gradually an acceptance of the posterior approach using the principles of Stoppa (Totally Extra Peritoneal technique, (TEP)) and facilitating the understanding and realization of the necessary parietalization, thanks to the magic of video images.

After this brief digression we can address the contribution of Franz Ugahary. At this period, it was interesting to note that exactly like Pelissier, Ugahary used Bassini and Lichtenstein as basic techniques, and Stoppa-Wantz for complex cases. In Ugahary’s mind the idea was to precisely reproduce the unilateral Stoppa (Wantz technique) by minimally invasive and purely posterior route. Thus, the Grid Iron type technique was born in 1995, influenced by the seamstress talents of the author’s grandmother, perfectly explaining Ugahary’s specific way of unrolling a classic or even lightweight mesh through a small incision [57]. With his technique, Ugahary is the true founder of the minimally invasive and minimal open totally preperitoneal and totally posterior technique. Ugahary’s strength was to propose new surgical principles, small “grid-iron” incision and specific dissection technique with special valves, to succeed in reproducing Wantz’s technique, with the possibility of using any regular or lightweight flat mesh and above all, allowing a technique that can be carried out under local anesthesia on an outpatient basis. For Pelissier, it was the invention of the prosthesis which allowed the realization of the TIPP technique, while Ugahary created a new operating technique that could use the then available basic meshes. Initially also being a vascular surgeon and having extensive experience in the extraperitoneal approach to large vessels [70], Ugahary took his perfect knowledge of anatomy, and he was very close to René Stoppa and Georges Wantz particularly. Finally, it was Ugahary who succeeded Wantz’s project, which was to perform a unilateral Stoppa in outpatient settings. His technique was immediately adopted by Georges Wantz, a very renowned hernia surgeon in New York, in the same way as his personal technique, especially after a demonstration carried out by F. Ugahary at the medical Hospital-Cornell Medical Center New York [70].

In 1997 René Stoppa, a close friend of Wantz and who directly inspired his technique, had visited Franz Ugahary in Tiel, Netherlands, who then immediately praised his technique as we can read in this letter addressed to F. Ugahary on 31 October 1997 [71]. “… I appreciated the principles of your operation: a posterior approach of Fruchaud myopectineal hole, a large piece of prosthetic mesh, your trend toward minimization of the wall surgical aggression (mini and grid-iron incision) … For me, your technical proposition seems to take place between Nyhus’ or Wantz’ operations through supra-inguinal incisions on one hand, and ours on the other hand … For transmitting, publishing, and teaching your technique, I suggest that you accurately describe every step and guide-marks. Mentioning pitfalls and errors are also a well-advised pedagogical precaution. Don’t forget that you are a gifted skilled surgeon, compared to many colleagues … ” The last sentence was loaded with meaning: is the technique easily reproducible? Stoppa asked me the same question directly while I was presenting the technique to the French academy of surgery in 2004 [72].

The first step of the original technique is the 3 cm incision above the deep inguinal ring as a McBurney incision without the incision of the peritoneum (Grid iron); the huge dissection in the preperitoneal space, typical for the Ugahary technique with different sizes of atraumatic retractors; the reduction of a medial sac, if any; the parietalization of the cord with a dissection of a lateral sac, if any; the checking of the femoral and obturator areas; the use of a 15 by 10 cm regular flat mesh or a lightweight mesh unrolled in the dissected space. No mesh fixation needed. No suture on the musculo fascial plane (transversalis fascia) [57].

In 2000, I was informed of the existence of the Kugel technique [73, 74] which was not yet available in Europe. At that time, I used to operate groin hernias usually with the endoscopic TAPP technique. I asked Stoppa for his opinion on the Kugel technique, He informed me that he knew the technique and had spoken twice with Kugel and without further comment he quickly advised me to visit F. Ugahary … After two short stays in Tiel (Netherlands), and the time necessary to assemble specially manufactured (and patented) instrumentation, I was able to carry out the technique in 2001. It quickly and definitively replaced my TAPP endoscopic technique. I preferentially used the original Ugahary technique between 2001 and 2011 for more than 1,000 hernia repairs with good results. A prospective study on the first 300 operated hernias has been published [72] by the French National Academy of Surgery in 2004. It showed the good results of the technique in terms of recurrence and chronic pain.

The unrolling of the flat mesh through the small incision according to the initial Ugahary technique appeared difficult to reproduce for many colleagues. To successfully carry out the intervention through a 3 – 4 cm incision, it was also necessary to have experience in the dissection of the pre-peritoneal spaces, to be familiar with the parietalization of the spermatic cord, to know how to handle the flexible parietal prostheses (flat polyester or polypropylene mesh, lightweight meshes … ) available at this period and to have the appropriate instrumentation (specific valves). For all these reasons, most surgeons still preferred the Lichtenstein technique, with less frequently endoscopic techniques, and while a few surgeons successfully used Ugahary’s principles, there were not many. I remained very motivated because I thought i had a very promising technique: on the one hand the good principles of a pure posterior preperitoneal approach: on the other hand, its resolutely minimally invasive nature, and finally with these very promising results in some hands. And even after having organized workshops in Cagnes sur Mer and after having presented the technique and its results in all directions (Congresses, French Academy of Surgery, publications) [72, 75, 76], it still appeared difficult to understand for many colleagues and therefore difficult to reproduce.

But as we detailed above, with the same state of mind (except that it is not a pure posterior route), we were joined by the TIPP technique. Following the ingenuity of Edouard Pelissier, it was easier to manipulate the prosthesis specifically created to be placed from the front in the preperitoneal space, which was very difficult to do with prostheses used endoscopically or with other prostheses available at this period.

**FIGURE 15:**
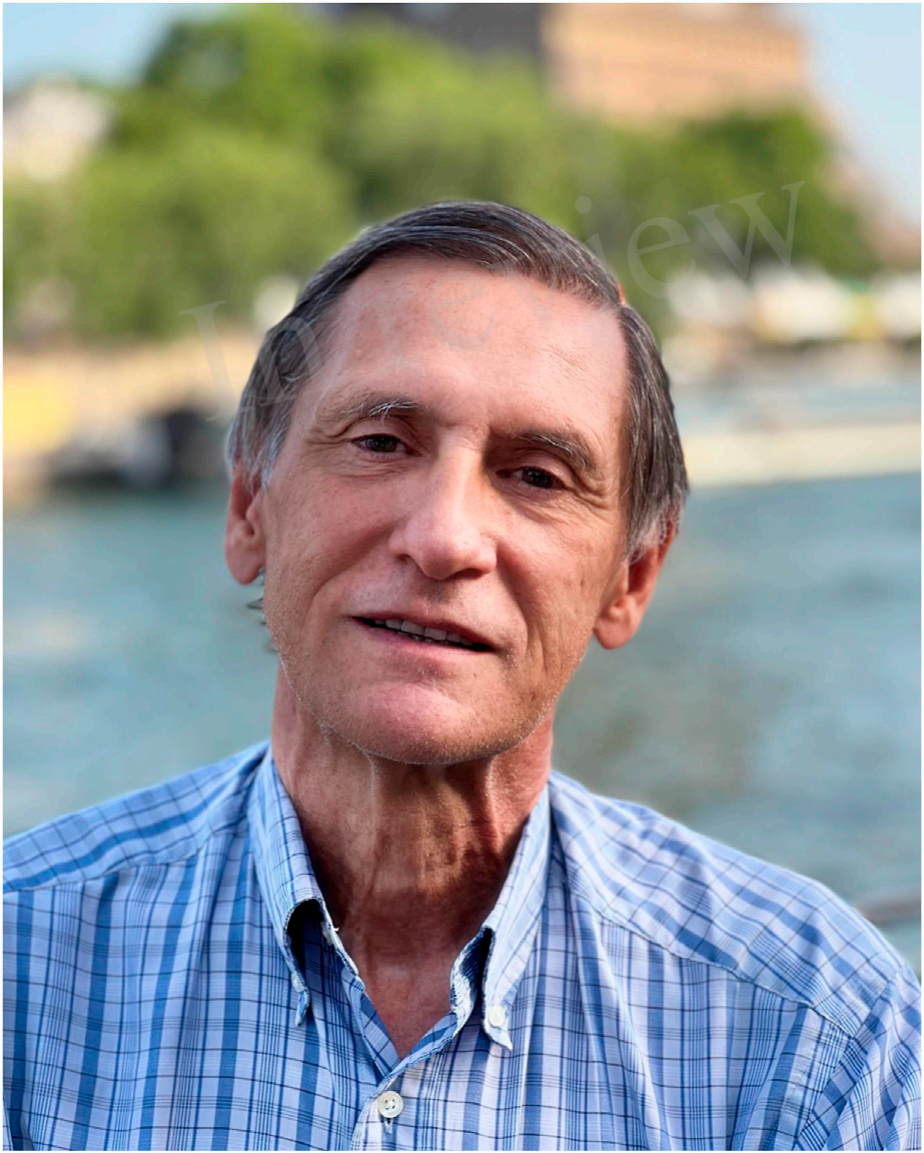
Marc Soler.

So, I had the idea (Figure 15) of combining some principles of Ugahary with others of TIPP. I visited Frederik Berrevoet, and Steven De Gendt in Ghent University Hospital in Oct 2007, and Jean François Gillion and Jean Michel Chollet in Antony Private Hospital near Paris to see the TIPP technique in some of the best hands. I carried out this project in 2011. The MOPP technique follows most of the steps of the TIPP technique: from the incision (which is somewhat reduced), the passage at the level of the inguinal canal through the deep inguinal ring, to the spreading of a large prosthesis in the preperitoneal space, with the specific method of Ugahary: dissecting the planes using different sizes of dissectors and retractors. This particularly atraumatic technique eliminates the need for any haemostasis procedures in the deep planes.

The innovation hinges on the identification of the transversalis fascia (TF) during two pivotal steps: The TF covers the deep inguinal orifice. Recognizing it at this juncture initiates the entry into the preperitoneal space, paving the way for preperitoneal dissection. The TF also constitutes the internal spermatic fascia. Identifying and severing it systematically commences the parietalization of the cord elements. Rationalizing these two essential steps for placing a large prosthesis in the preperitoneal space makes the technique more easily reproducible and teachable. The publication with the help of the specific French database (only used by parietal specialists) have shown very good results on the technique in the hands of the author [77, 78]. More long-term results and a very precise presentation of all the stages of the technique were published in 2024 [79]. The results concerning 1,401 patients show a very low recurrence rate and chronic pain.

Willem Akkersdijk has also tackled the challenge in 2006 with the Trans Rectus sheath Pre-Peritoneal (TREPP) technique [80], a sutureless technique in the same spirit as the Ugahary technique but using the TIPP (Pelissier) prosthesis! TREPP added a significant advantage to the open and minimally invasive preperitoneal approach. It is a perfectly codified [81], with 9 steps, pure posterior approach therefore leaving the anterior planes completely untouched unlike the TIPP and MOPP techniques which are techniques that open the inguinal canal. The TREPP technique is perfectly suitable for nearly all kind of groin hernias, including some of recurrent ones [82–84]. As W. Akkersdijk said [85], “TREPP was developed in the era of endoscopic surgery. The preperitoneal space had gained popularity and the upstream principle was advocated as a logical way to keep the mesh in the right position, even without fixing it and to minimize the chance of recurrences. TREPP has not always been called the same. In the beginning it was called the rectus sheath approach. The absence of a posterior rectus fascia was one of the reasons this route was chosen to reach the preperitoneal spaces just beneath the lateral edges of the rectus muscle. Compared to other open preperitoneal techniques, Ugahary, Pelissier, Rives-Stoppa, with the TREPP technique, the dissection was minimized and the view on the internal ring was optimized by the point of entrance of the preperitoneal space.” W. Akkersdijk insists on the fact that “muscle tension may also cause problems in the creation of the preperitoneal space. Optimal muscle relaxation can be reached by spinal anesthesia or like in endoscopic groin surgery, general anesthesia.”

It seemed obvious to all the pioneers of the new open approach that this minimal open route was much less invasive than endoscopic techniques known as minimally invasive techniques (MIS)! But these were only expert opinions without published studies with a high level of evidence…

It is only in recent years that the literature has provided data about TIPP and TREPP, with comparisons to other techniques and has shown good results of this third way of operating on inguinal hernias. However, drawing parallels to other preperitoneal techniques suggests that MOPP’s outcomes might align with other open or endoscopic methods such as TREPP, TEP and TAPP. Hurel and colleagues [86] support this assumption in their conclusion from a recent propensity score matching analysis comparing 1-year postoperative chronic pain using Lichtenstein, TIPP (including MOPP), TAPP and TEP techniques. Their findings highlight Lichtenstein’s clear disadvantage and an indistinguishable difference between TIPP (including MOPP), TAPP and TEP. To further explore the potential benefits of the open preperitoneal approach, consider this study by M. Reinhorn and colleagues [87] which emphasizes the potential benefits of open posterior mesh placement (TREPP) over endoscopic repair in terms of short-term Quality of Life (QoL) and seroma formation, with equivalent hernia recurrence rates. Agarwal et al. [88] show the advantages of TREPP/MOPP over Lichtenstein regarding patient-reported QoL, sustained for a year, and reduced opioid intake 30 days post-surgery. Zwols [89] and Koning [90] also highlight the superiority of preperitoneal techniques over the Lichtenstein method. J.L. Faessen [91] shows in pilot study that TREPP is comparable to TEP and Lichtenstein in terms of recurrence rates, chronic post-operative inguinal pain, and clinically significant adverse events.

## Conclusion

Ongoing studies expert are published in the same JAWS special issue provide additional information regarding the treatment of scrotal hernias using the MOPP technique [92], as well as another study comparing the results of the treatment of scrotal hernias using TIPP/MOPP versus Lichtenstein and endoscopic techniques [93]. I hope that this article and the special issue dedicated to the modern preperitoneal minimally invasive route will open lots of eyes. New studies, randomized, or as I think, using smart data from specific databases, must confirm the advantages or equivalences of these techniques compared to the other two groups. It should be interesting to highlight the advantages due to the minimally invasive nature, making it possible to operate on more complex hernias and the most fragile or elderly patients, using a large preperitoneal prosthesis and thus avoiding the Lichtenstein technique more commonly used in these patients. But, at this moment, this is still only the idea of a small percentage of surgeons. As Usher experienced as reported by Read, as Rives experienced and expressed to me, as Fruchaud experienced as reported by Stoppa, innovators often struggle to move their ideas forward and bring them to fruition. This is perhaps what is happening to the promoters of minimally invasive preperitoneal surgery who for nearly 30 years have been campaigning for this third way, having the virtues of the great principles currently accepted and the virtues of less invasiveness, and more economical and more ecological. Time will tell whether this path will have its place or whether it will only delay the use of new technologies for all.
